# Supplementary material for: Histone divergence in trypanosomes results in unique alterations to nucleosome structure
Source: Nucleic Acids Res. 2023 Jul 10;51(15):7882–99. doi: 10.1093/nar/gkad577 (PMC10450195; doi:10.1093/nar/gkad577)
Supplement: gkad577_Supplemental_Files [file gkad577_supplemental_files.zip › ALL Supplementary Figures_a4_proof_correction_ref_added.pdf]

Histone divergence in trypanosomes results in unique alterations to nucleosome structure

SUPPLEMENTARY FIGURES

Gauri Deák[1], Hannah Wapenaar[1], Gorka Sandoval[1], Ruofan Chen[1], Mark R. D. Taylor[1], Hayden Burdett[1], James A. Watson[1], Maarten W. Tuijtel[1,2], Shaun Webb[1], Marcus D. Wilson[1]*

[1] Wellcome Centre for Cell Biology, University of Edinburgh, Michael Swann Building, Kings Buildings, Mayfield Road, Edinburgh, EH9 3JR, UK
[2] Current address: Department of Molecular Sociology, Max Planck Institute of Biophysics, Max-von-Laue-Straße 3, 60438 Frankfurt am Main, Germany
*Correspondence should be addressed to marcus.wilson@ed.ac.uk

Supplementary Figure S1

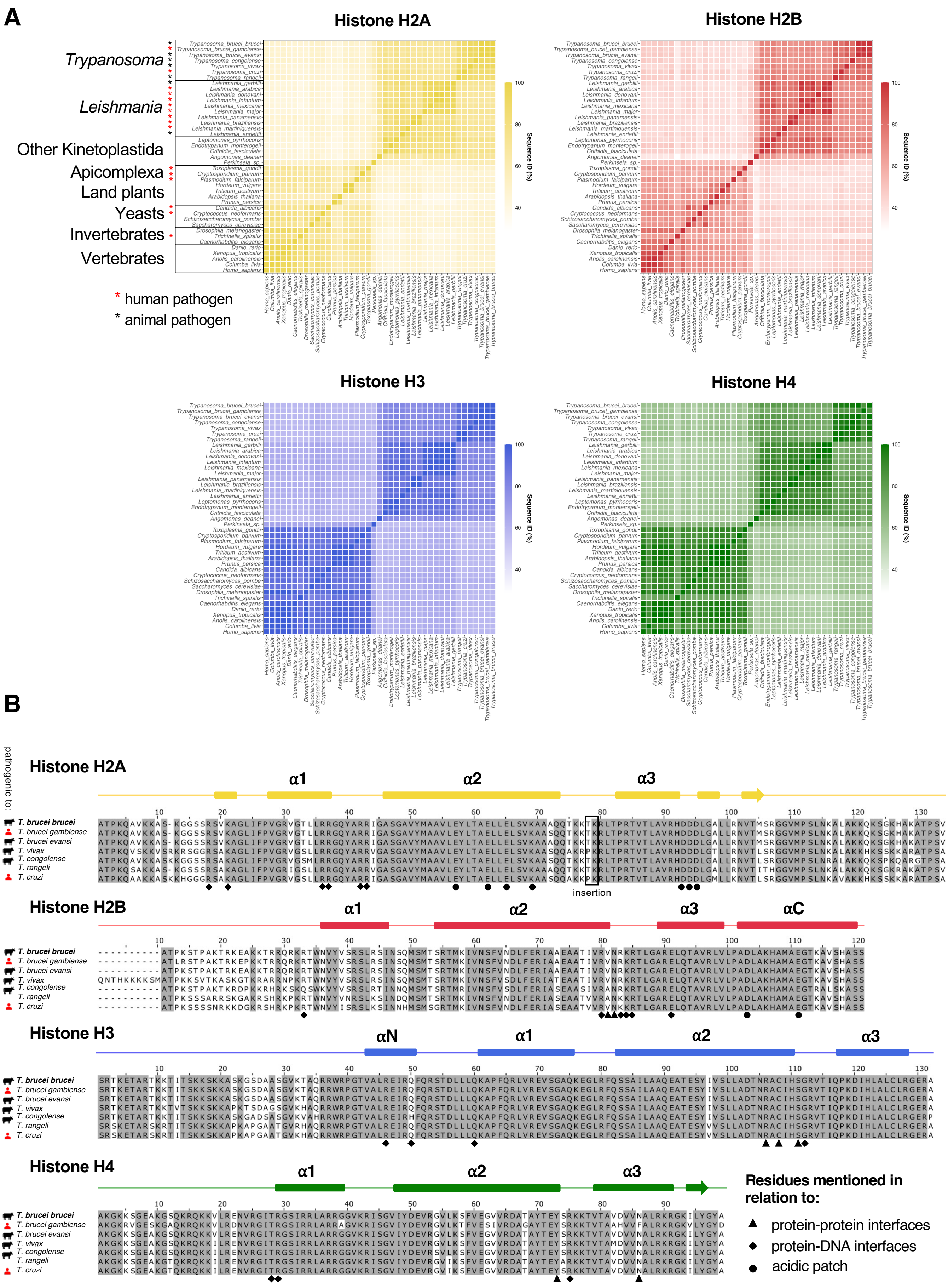

A. Pairwise sequence identity (ID) matrices of histones from various eukaryotic species. Groups of species that are present in all four matrices are outlined in the panel for histone H2A. Human and animal pathogens are marked with red and black asterisks respectively.

B. Multiple sequence alignment of histone sequences from *Trypanosoma* species with annotations highlighting residues that are mentioned in relation to histone-histone interfaces, histone-DNA interfaces or the acidic patch in this study. Secondary structure annotations are based on the *T. brucei* NCP model presented in this study.

Supplementary Figure S2: Quality control of *in vitro* reconstituted NCPs and tetrasomes

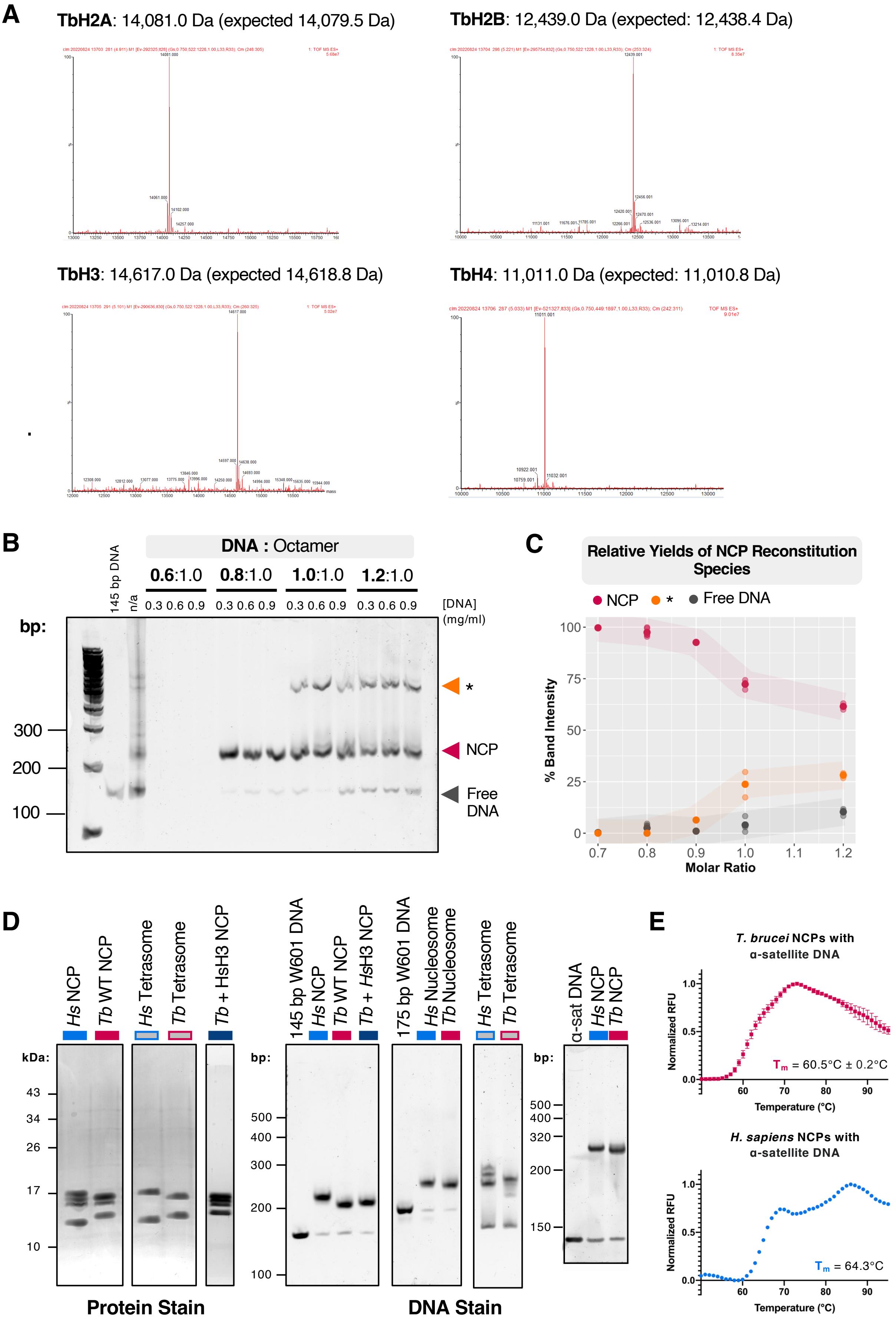

A. 1D Intact mass spectrometry analysis of each *T. brucei* histone showing their deconvolved mass profile. **B**. A range of DNA:octamer molar ratios tested to optimize wrapping of *T. brucei* histone octamers with different concentrations of Widom 601 145 bp DNA (wrapped NCPs = pink, free DNA = grey, unknown higher molecular weight species (‘*’) = orange). **C**. Quantification of relative band intensities from optimization experiments such as **B**. **D**. Gels showing NCPs reconstituted *in vitro* with Widom 601 145 bp DNA and alpha satellite 147 bp DNA, nucleosomes reconstituted with 175 bp FAM-labelled DNA, and H3-H4 tetrasomes consisting reconstituted with 145 bp Widom 601 DNA. On the left, SDS-PAGE gels show equivalent histone distribution. On the right, native polyacrylamide gels show the shift in electrophoretic mobility of DNA when wrapped into NCPs/nucleosomes/tetrasomes. **E**. Thermal denaturation assays of *T. brucei* (top) and *H. sapiens* (bottom) NCPs wrapped with 147 bp alpha-satellite DNA (melting temperatures (T_m) are indicated).

Supplementary Figure S3: Structural and biophysical characterization of the *T. brucei* NCP

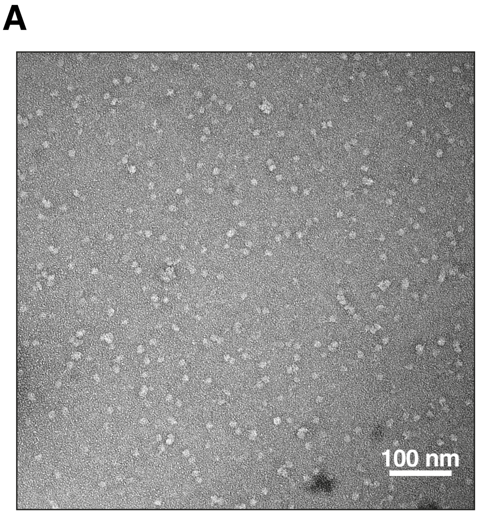

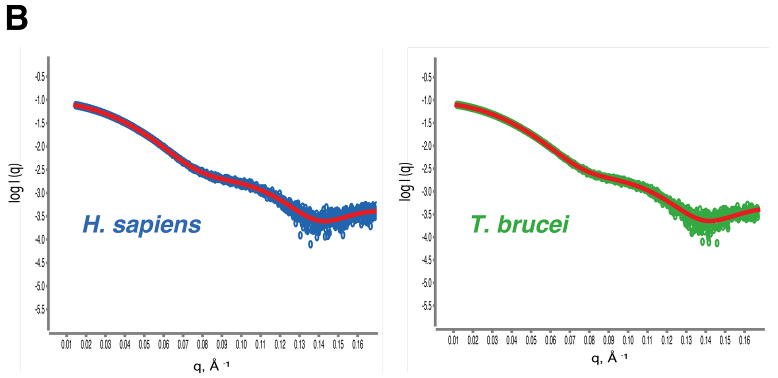

	Homo sapiens NCP	*Trypanosoma brucei* NCP
R_g (Real, Guinier)	42.3 Å	42.1 Å
(Reciprocal, Pr)	43.6 Å	43.1 Å
D_{max}	120 Å	126 Å

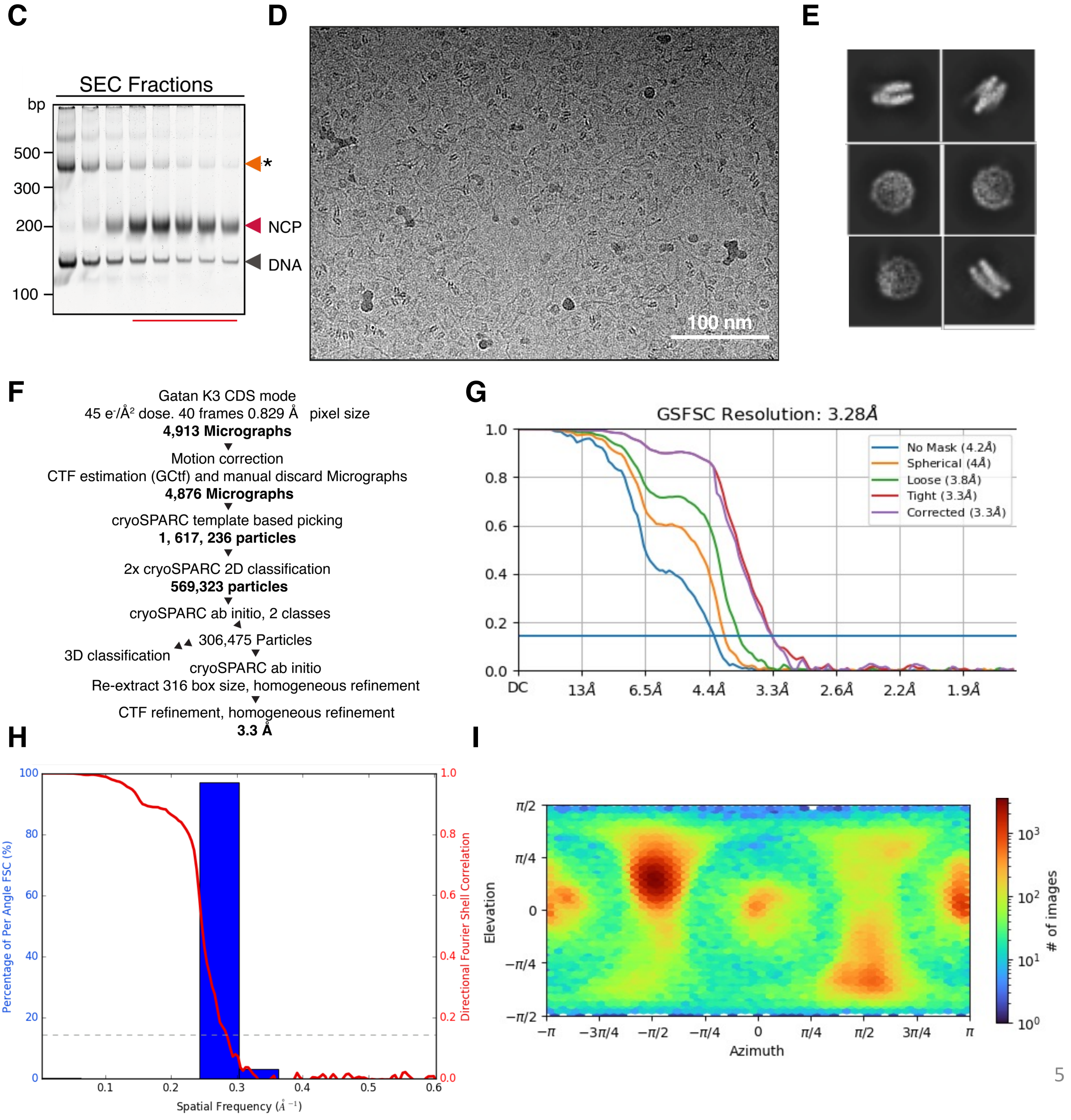

A. Representative negative stain EM micrograph of the T. brucei NCP, showing dispersed primarily ~10 nm top view images. **B**. (top) SAXS scattering profile of and *H. sapiens* (left, blue) and *T. brucei* (green, right) with scattering model fit. (bottom). Table summarizing the radius of gyration (Rg) and maximum dimension (Dmax) calculated from the data using both Porod-Debye (Pr) distribution or normalised Guinier analysis. Rg and Dmax values correlate well suggesting that the averaged size between NCPs is similar. **C.** Native polyacrylamide gel of glutaraldehyde crosslinked NCPs, separated by size exclusion chromatography (SEC) form over crosslinked species, aggregate band (orange) and free DNA (grey). Pooled fractions for cryo-EM grid preparation are indicated with red line. **D**. Representative cryo-EM micrograph of the *T. brucei* NCP. **E**. Examples of 2D class averages obtained during image processing. **F**. flowchart describing cryo-EM image processing pipeline. **G**. Gold-standard Fourier shell correlation (GS-FSC) curve for final map, including unmasked and masked curves.The blue line corresponds to 0.143 threshold. **H.** Three dimensional FSC (1) for final map showing global FSC curve (red) and overlap of histogram of directional FSC with the major peak correlating with the global resolution estimate. **I.** Euler angle distribution plot of all particles used in the final map. Despite some preferred orientations (red on heat map) no anisotropy in model was observed (h).

Supplementary Figure S4: Histone secondary structure and alignment in the *T. brucei* NCP.

A

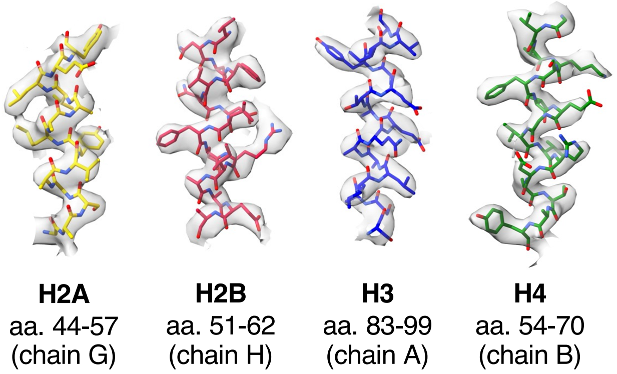

B

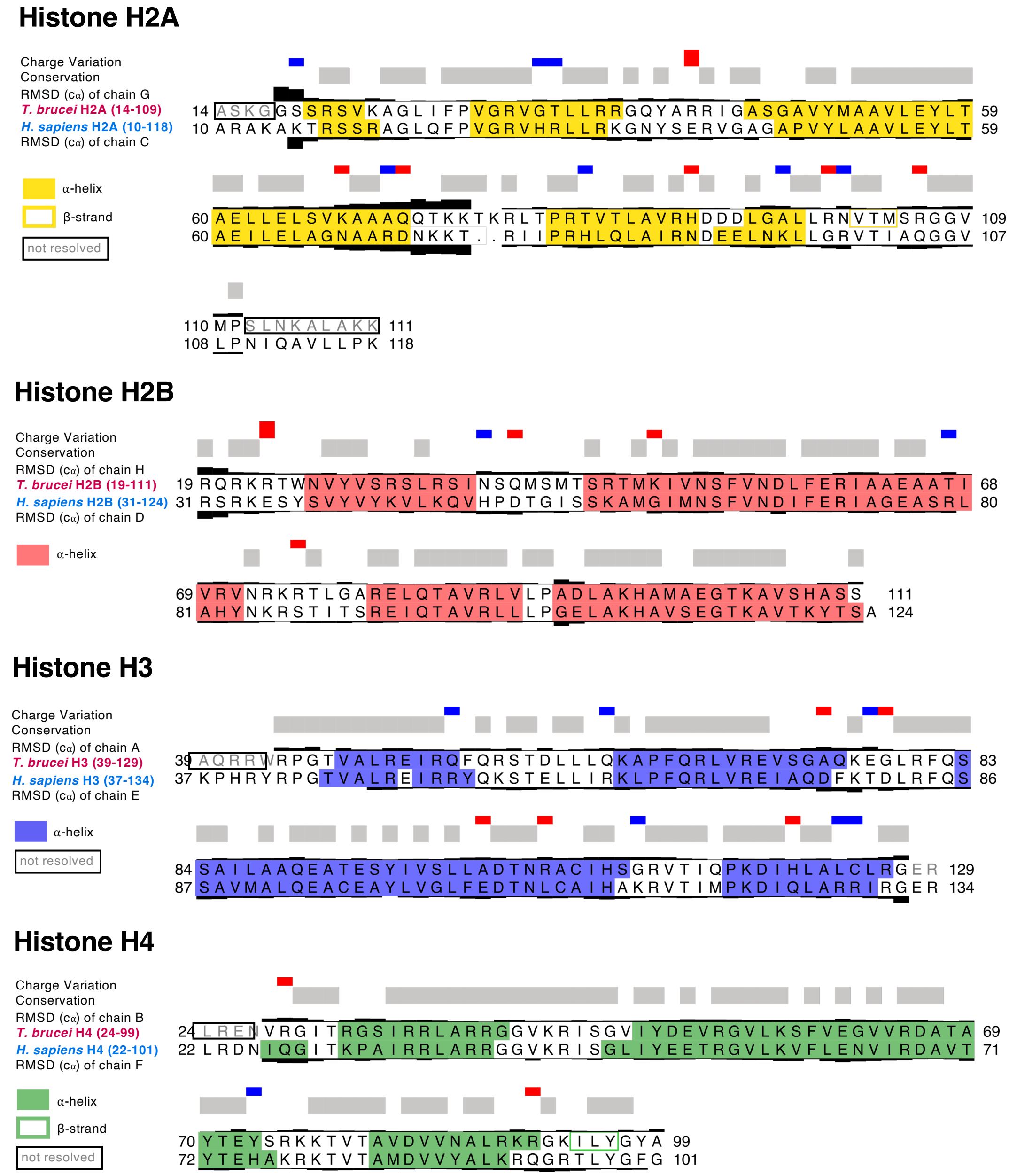

A. Representative images showing the EM density and model building for each *T. brucei* histone**. B.** Pairwise structural alignment of histones from *T. brucei* (our model) and *H. sapiens* (PDB: 7XD1) (2)*.* **Charge variation** highlights changes in *H. sapiens* sequences compared to *T. brucei*, where blue = change to a positively charged residue, red = change to a negatively charged residue, and the height of the bar represents the magnitude of the change. **Conserved residues** are indicated in grey and the **RMSD values** of c_{α} atoms in both chains are shown as black bars, where a larger bar indicates higher RMSD.

Supplementary Figure S5: **Weakened DNA binding in the *T. brucei* NCP occurs due to alteration of local protein-DNA contacts.**

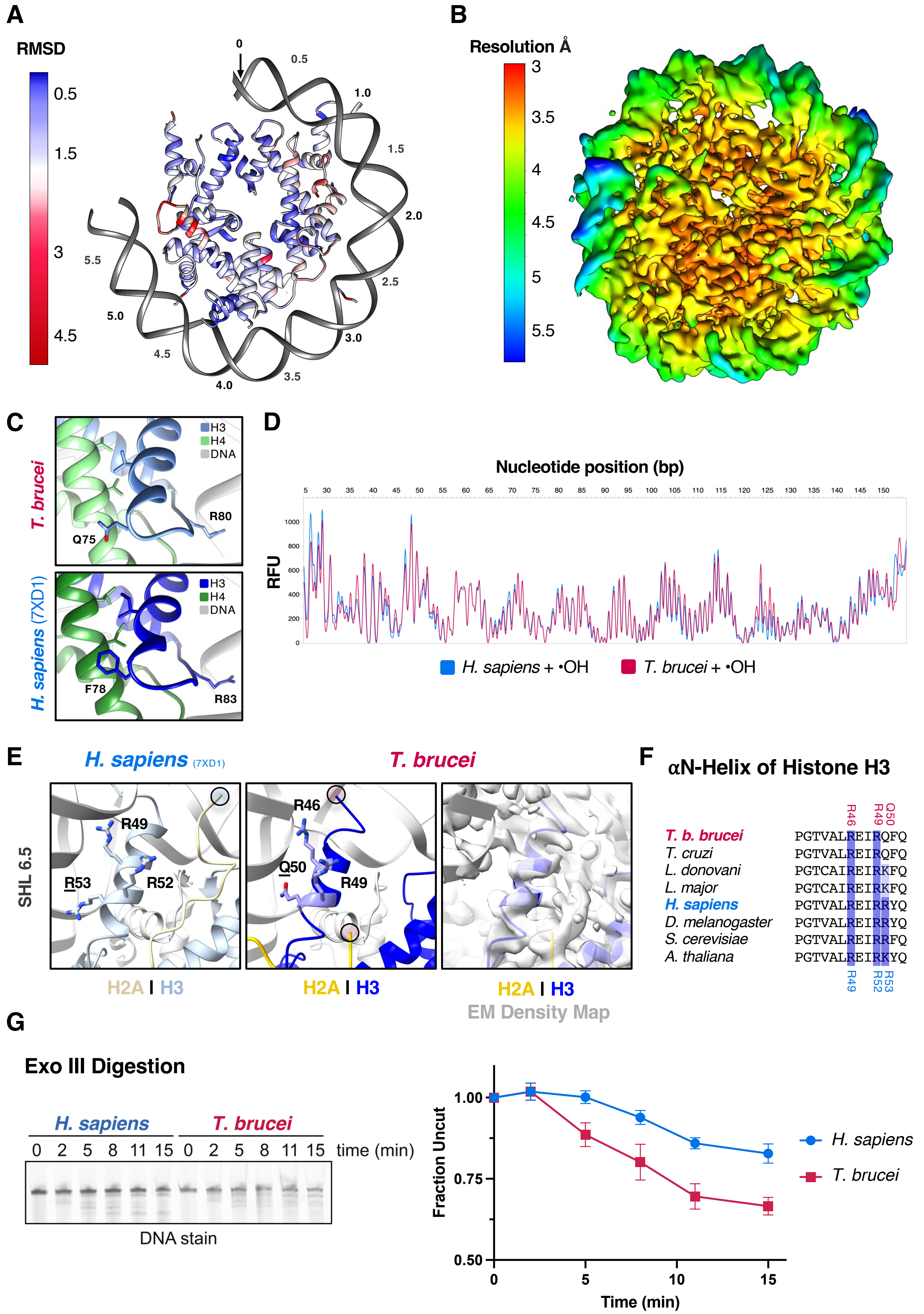

A. Lateral view *of T. brucei* NCP model, histones coloured according to root mean square deviation (RMSD) between C_α atoms in this structure and the structure of the *X. laevis* NCP (PDB: 3LZ0) (3). Only one side of the NCP is shown for clarity. **B**. Cryo-EM density *of T. brucei* NCP coloured according to local resolution, estimated in CryoSPARC. The core of the NCP is highly ordered while the DNA ends are flexible due trypanosome-specific histone sequence alterations. **C.** Close-up comparison of the packing of histones H3 and H4 in the H3 elbow region of *T. brucei* and *H. sapiens* NCPs (see Figure 2B). **D**. Hydroxyl radical (•OH) footprinting assay on *H. sapiens* and *T. brucei* nucleosomes wrapped with Widom 601 175 bp FAM-labelled DNA (detectable fragment size range 25 bp – 155 bp). ~10 bp periodicity of protected residues corresponding to histone-DNA interactions can be seen for both *T. brucei* and *H. sapiens* NCPs, suggesting that overall DNA register is maintained. **E**. Comparison of the N-terminal end of H3 and the C-terminal end of H2A at SHL6 in *H. sapiens* (left) and *T. brucei* (right), including weaker EM density (far right). Circles indicate the end of buildable density on histone tails. **F**. Multiple sequence alignment of the αN helix of histone H3 with DNA. Residues that were previously identified as crucial for contacting DNA (4,5) are highlighted. **G**. Denaturing urea gel (left) and quantification of the disappearance of the 145 bp DNA (right) from an Exonuclease III digestion assay of intact NCPs at indicated timepoints.

Supplementary Figure S6: Divergent interfaces in the *T. brucei* NCP dictate the formation and stability of chimeric histone assemblies

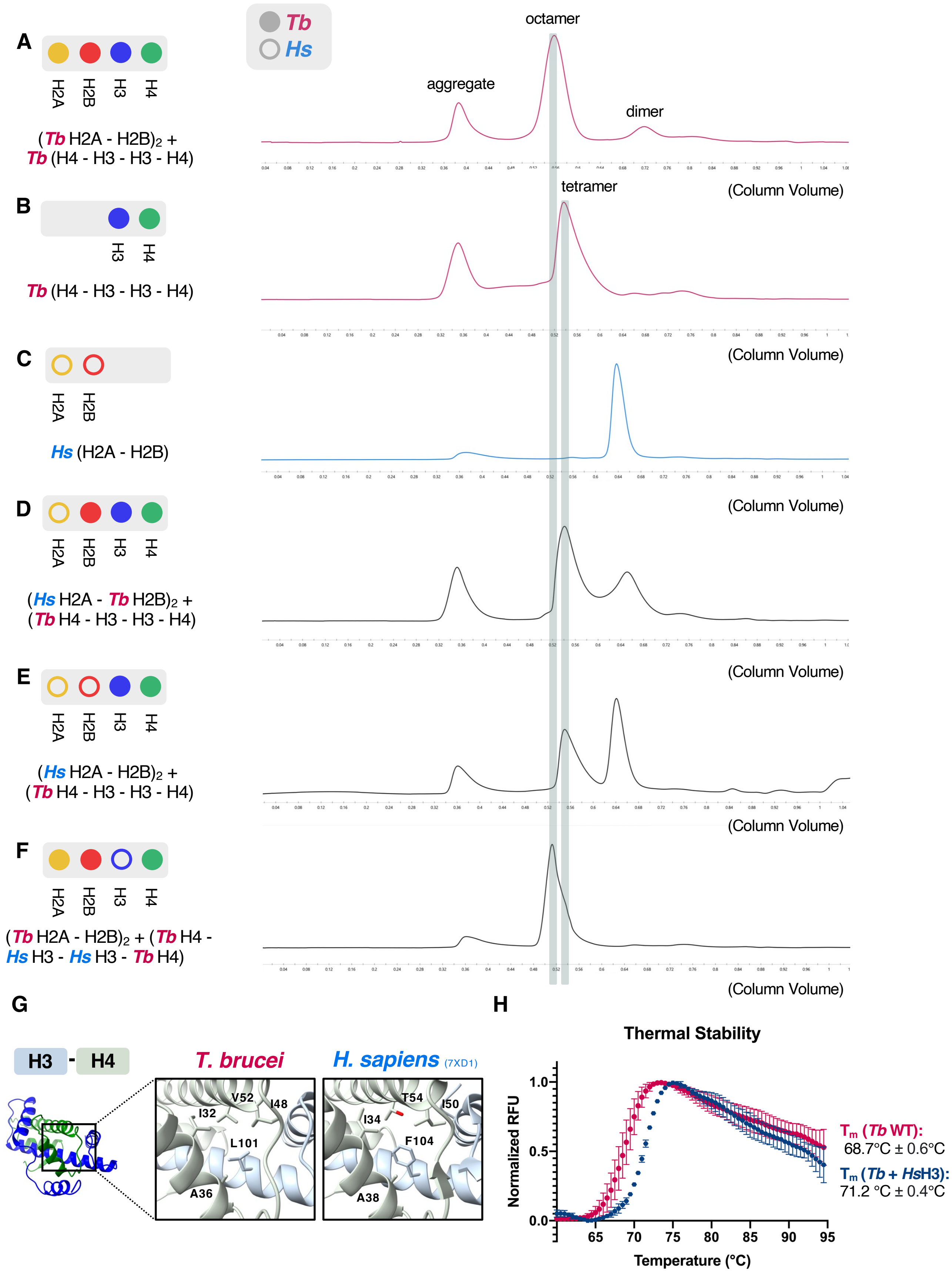

Size exclusion chromatograms of various histone complexes after the refolding step of octamer/tetramer/dimer assembly showing lack of assembly for some chimeric octamers. Expected elution volume for the different species is highlighted. **A.** *T. brucei* octamers (used for "Tb WT" NCPs); **B.** *Tb* H3-H4 tetramers; **C.** *Hs* H2A-H2B dimers; **D.** *Tb* H2B, H3, and H4 *Hs* H2A; **E.** *Tb* H3-H4 tetramers with *Hs* H2A-H2B; **F.** *Tb* H2A, H2B, and H4 with *Hs* H3 (used for *"T. brucei* + *Hs*H3" NCPs). **G.** Comparison of the H3-H4 interface in *T. brucei* and *H. sapiens.* **H.** Comparison of thermal denaturation curves from *T. brucei* WT and *T. brucei* + *Hs*H3 NCPs (the only chimeric NCP that could be generated).

Supplementary Figure S7: Altered DNA contacts in the T. brucei NCP lead to reduced DNA binding and instability.

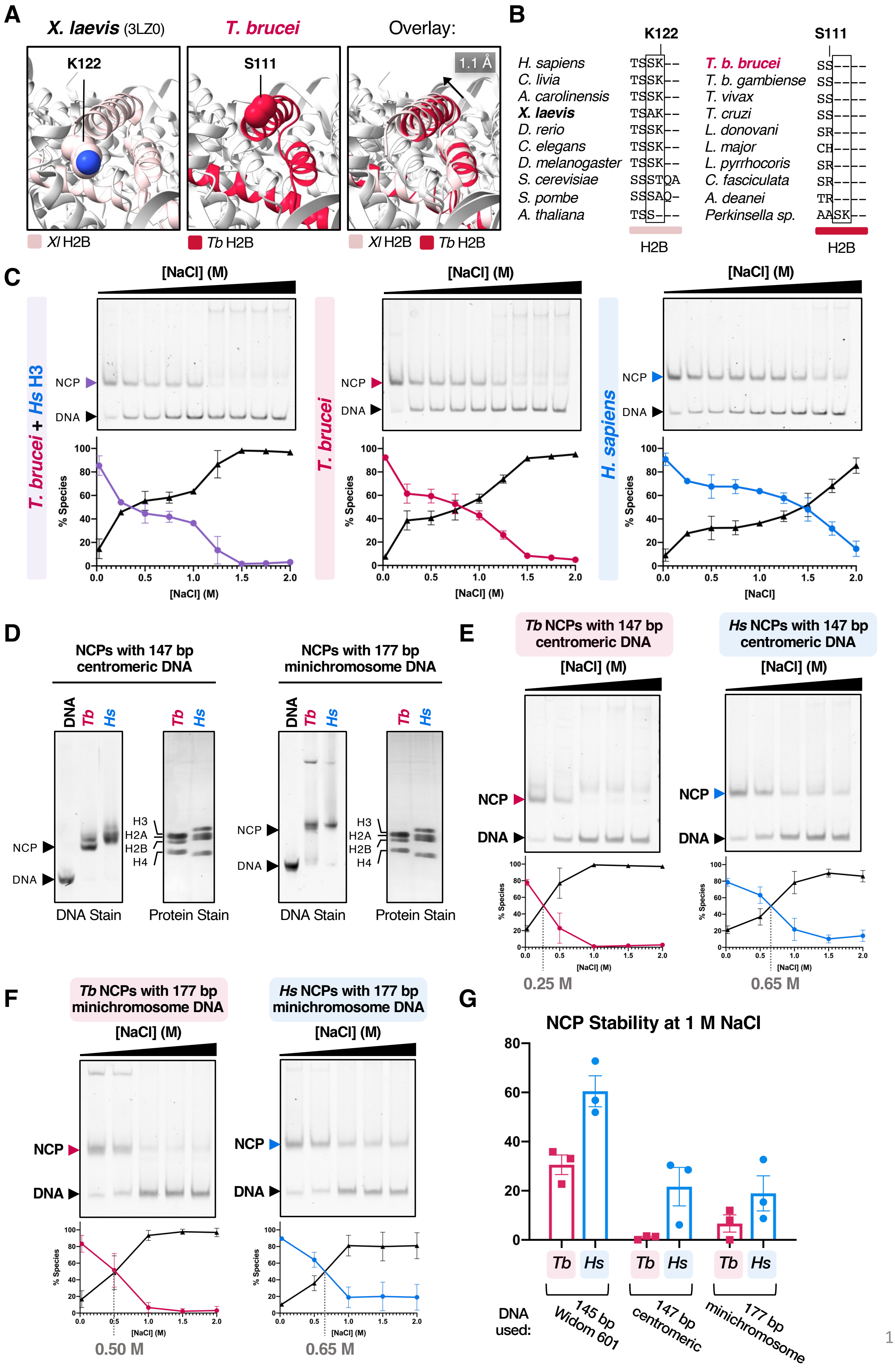

A. Comparison of the C-terminal helix of histone H2B in *X. laevis* (left)(3) and *T. brucei* (right), highlighting the lack of a terminal lysine residue and altered packing in the *T. brucei* NCP (far right). **B.** Multiple sequence alignment of the end of C-terminal helix of H2B. H2B is shorter by two residues in kinetoplastids compared to most of the other eukaryotes. **C.** Native polyacrylamide gels stained for DNA comparing 145 bp Widom 601 DNA unwrapping in *T. brucei* + *Hs* H3 NCPs vs. *T. brucei* and *H. sapiens* NCPs over a broad range of NaCl concentrations. **D.** *T. brucei* and *H. sapiens* NCPs reconstituted with 147 bp centromere-associated repeat DNA and 177 bp minichromosome DNA shown in both their native (DNA stain) and denatured form (protein stain). **E.-F.** Native polyacrylamide gels stained for DNA showing DNA unwrapping in *T. brucei* and *H. sapiens* NCPs wrapped with the 147 bp centromere-associated DNA (**E**) and 177 bp minichromosome DNA (**F**) after incubation at different NaCl concentrations. The NaCl concentration at which free DNA overtakes wrapped NCPs is indicated in grey. **G.** Comparison of the percentage of NCP remaining at 1 M NaCl in NCPs wrapped with different DNA sequences.

Supplementary Figure S8: A cluster of positively charged residues drives increased DNA binding at SHL3.5 in the T. brucei NCP.

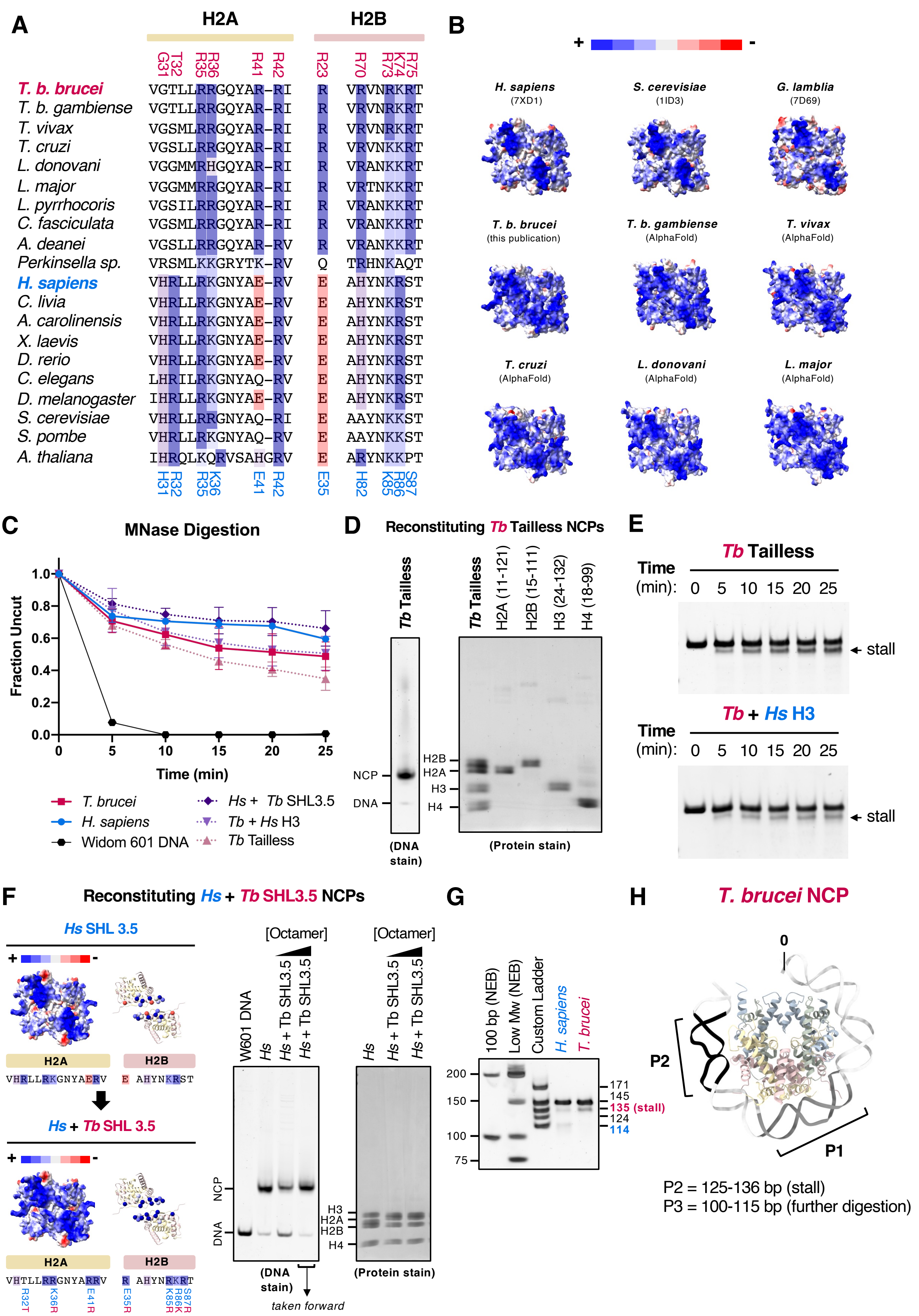

A. Multiple sequence alignment of key residues at the SHL3.5 interface, showing conservation of extra basic residues predicted in this region. **B**. Comparison of the interface at SHL3.5 in histone octamers from multiple species. Models were either created using AlphaFold2 (6) or taken from previously published structures and colored by surface electrostatics. **C.** Quantification of the loss of the full-length band in MNase digestion assays of various NCPs from three independent experiments each. **D.** Reconstitution of NCPs with tailless *T. brucei* histones and Widom 601 145 bp DNA shown in both their native (DNA stain) and denatured states (protein stain). **E.** MNase digestion assays of *Tb* tailless and *Tb* + *Hs*H3 NCPs showing retention of the stall band that is characteristic for *T. brucei* NCPs. **F. (left)** A schematic showing the effect of the H2A-H2B mutations in *H. sapiens* + *Tb* SHL3.5 NCPs on net positive charge at the SHL3.5 histone-DNA interface. **(right)** Concurrent reconstitution of *H. sapiens* NCPs and *H. sapiens* + *Tb* SHL3.5 NCPs with Widom 601 145 bp DNA at different molar ratios of DNA:histone octamer. **G.** Native polyacrylamide gel of MNase digestion products from *H. sapiens* and *T. brucei* NCPs after 30 min next to multiple DNA ladders to map the *T. brucei*-specific stall point. **H.** MNase 'stall points' at Peaks 1 and 2 (P1, P2 from Figure 5E in the main text) mapped onto the structure of the *T. brucei* NCP. Peak 1 is the major stall point and digestion occurs predominantly from one side of the NCP.

Supplementary Figure S9: The kinetoplastid acidic patch is highly divergent and extensive mutagenesis of the *T. brucei* acidic patch does not rescue binding to known interactors

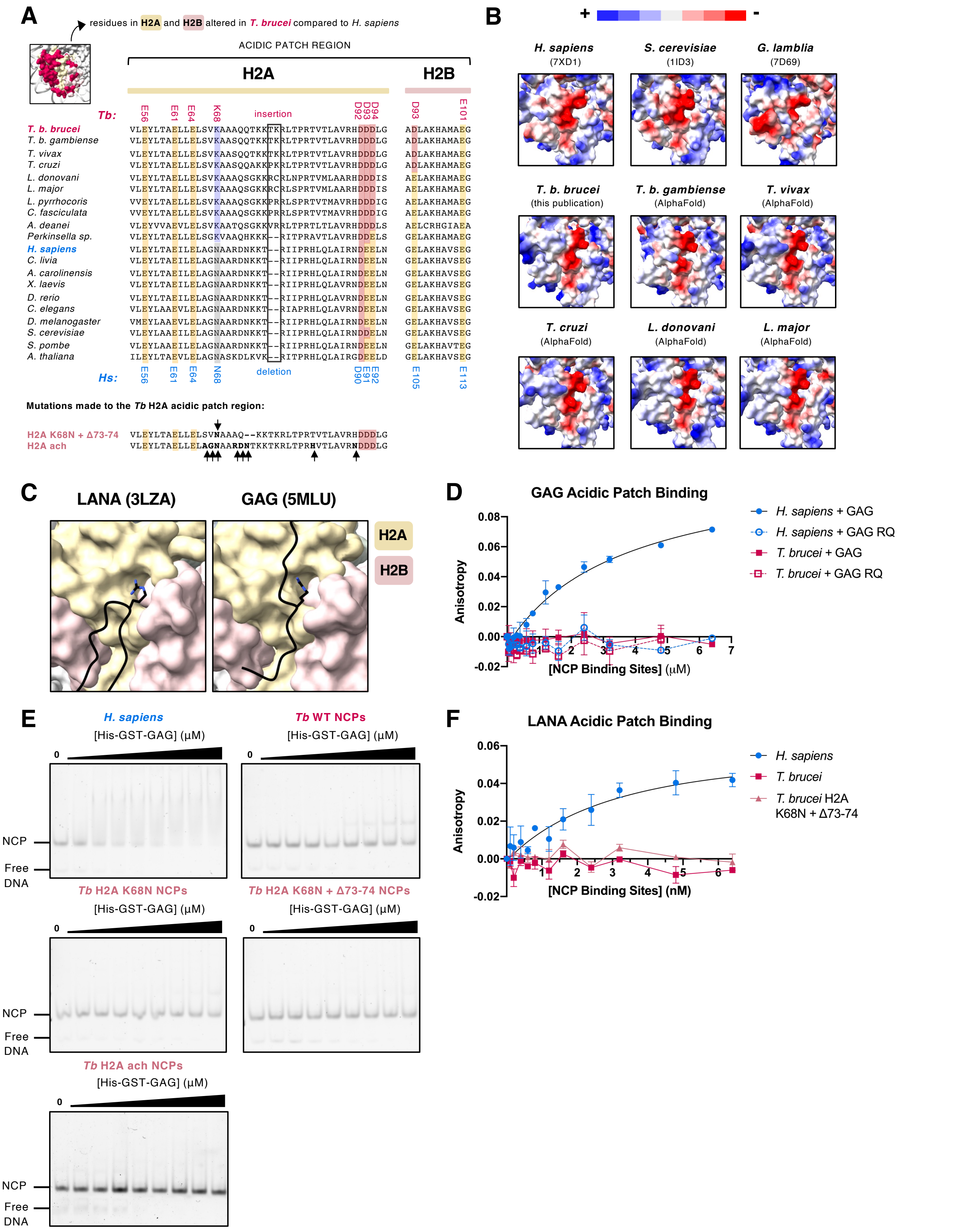

A. Extended multiple sequence alignment of acidic patch regions in H2A and H2B (see Figure 6A). Residues that are different in *T. brucei* H2A and H2B compared to *H. sapiens* are visualized on the structure of the *T. brucei* NCP on the top left. *Tb* H2A sequences containing 'humanizing' mutations that were used in part **E.** are shown below and indicated with black arrows. In **'H2A K68N + Δ73-74'**, *Tb* H2A-Lys68 is mutated to *Hs* H2A-Asn68 and two residues are deleted to remove the kinetoplastid-specific insertion. In **'H2A-ach'**, multiple mutations are made to residues surrounding the acidic patch by comparing sequence conservation in kinetoplastids and other organisms (S66A + V67G + K68N + A71R + Q72D + Q73N + T84H + H91N). **B.** Comparison of the acidic patch region in histone octamers from various species shown with surface electrostatics, either predicted using AlphaFold2 (6), from *T. brucei* (this study, see Figure 6), or from previously published NCP structures (2,7,8). **C.** Differential acidic patch binding modes of LANA (9) and GAG (10) peptides based on published crystal structures. **D.** Fluorescence polarization assay with a FITC-tagged GAG peptide and a mutated, non-binding GAG peptide ('GAG RQ') vs. *H. sapiens* and *T. brucei* NCPs. **E.** Electrophoretic mobility shift assays with His-GST-tagged GAG peptide incubated at increasing concentrations with various NCPs including *H. sapiens*, *T. brucei*, *T. brucei* with H2A K68N, *T. brucei* with H2A K68N + Δ73-74, and *Tb* H2A ach ([His-GST-GAG] (μM): 0, 0.2, 0.4, 0.6, 0.8, 1.0, 1.2, 1.4, 1.6; K_D ~ 3.5 μM). **F.** Fluorescence polarization assay with a FITC-tagged LANA peptide comparing LANA binding to *T. brucei* NCPs mutated at H2A K68N + Δ73-74 vs. *T. brucei* and *H. sapiens* NCPs (binding to non-mutated NCPs also shown in Figure 6D).

Supplementary Figure S10: The surface of *T. brucei* NCP likely serves as a dynamic interaction site for chromatin binders

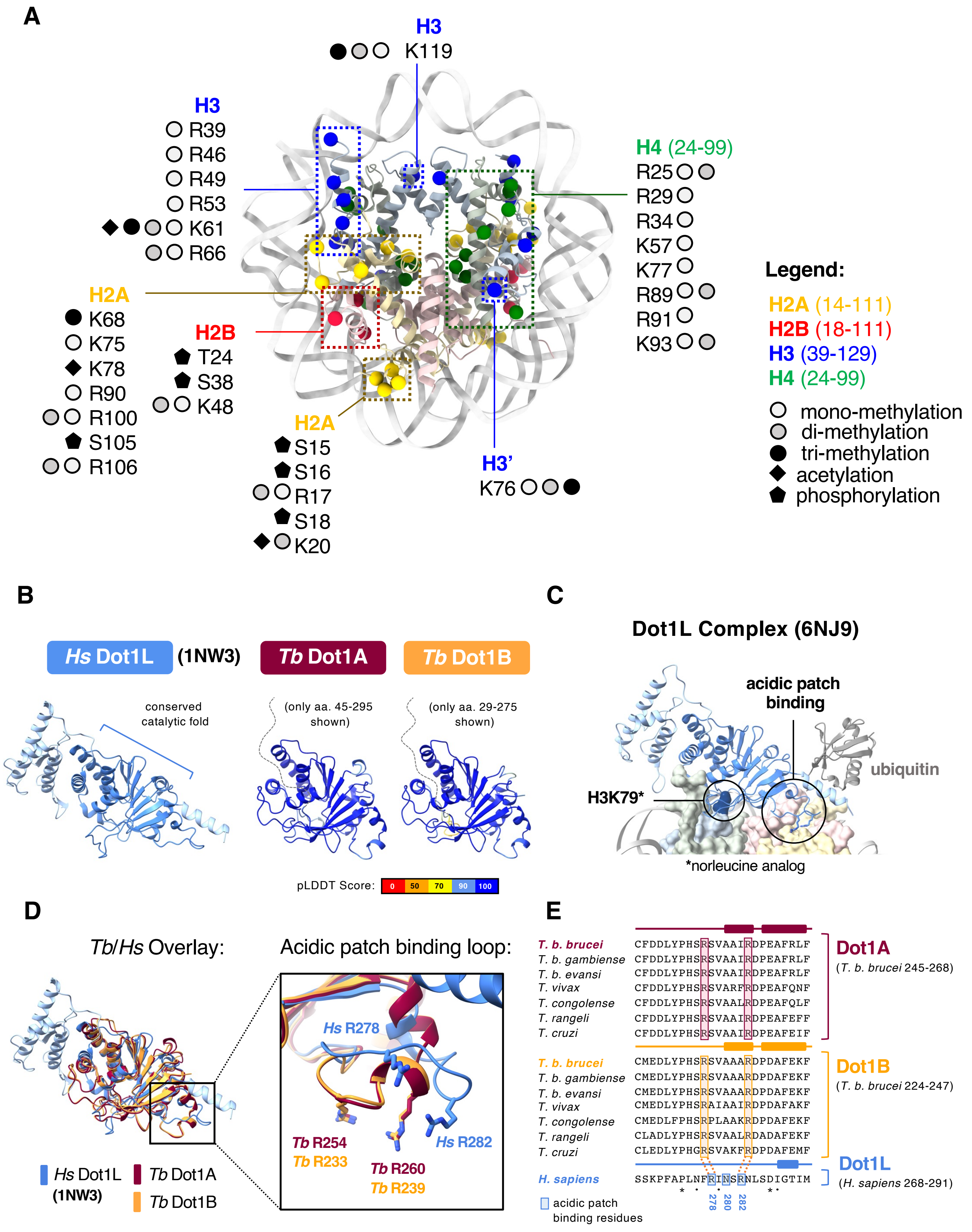

A. Previously identified histone post-translational modifications (11,12) mapped onto the structure of the *T. brucei* NCP. **B**. Conservation of the catalytic fold of *H. sapiens* Dot1L (13) and *T. brucei* Dot1A/Dot1A predicted by AlphaFold2 (6). Predicted structures are coloured by their pLDDT confidence score. **C**. *H. sapiens* Dot1L binding to the nucleosome by contacting its substrate H3-Lys79, the acidic patch, and ubiquitin (14). Histone ubiquitylation does not seem to be conserved in *T. brucei*, and neither is the ubiquitin interacting region. **D**. Overlay of *H. sapiens* Dot1L and *T. brucei* Dot1A/B and a magnified view of the acidic patch binding loop characterized in *H. sapiens* (13,14) **E**. Multiple sequence alignment of the putative acidic patch binding region in Dot1A/B sequences from the *Trypanosoma spp.* This loop is highly divergent compared to the sequence of the *H. sapiens* Dot1L region, but conserved in the *Trypanosoma spp.* (asterisk = identical, dot = similar amino acid). Residues in Dot1L that were shown to bind to the acidic patch (R287, N280, and R282) (14,15) are highlighted in blue. The potential arginine equivalents in *T. brucei* Dot1A and Dot1B are highlighted in pink and orange, respectively. The asparagine is lacking and is replaced by an alanine in a cluster of small hydrophobic residues that appear to increase the alpha-helical propensity of the loop. Secondary structure elements for each alignment are shown above. These alterations are consistent with the extensive structural divergence of the trypanosome acidic patch.

Supplementary Movie Legends

Supplementary Movie S1: Overview of the cryo-EM density map and built model of the *T. brucei* NCP

The map is coloured according to density attributed to histones (H2A = yellow, H2B = red, H3 = blue, H4 = green, DNA = grey) using the colour zone tool in ChimeraX (16).

Supplementary Movie S2: Comparison of the shape of the *H. sapiens* and *T. brucei* NCPs

Overview showing the oval shape of the *T. brucei* NCP morphing into the *H. sapiens* NCP (PDB: 7XD1) (2), followed by a zoomed view of the sites of compression in the *T. brucei* NCP (SHL6 and SHL2). Created using the morph feature in ChimeraX (16).

Supplementary Table S1: Data collection parameters, processing information, and model validation metrics from cryo-EM structure determination of the *T. brucei* NCP.

Parameter	*T. brucei* NCP
**Data Collection**	
Microscope	TFS Titan Krios
Detector	Gatan K3
Acceleration voltage (kV)	300
Number of micrographs	4913
Frames per micrographs	40
Exposure time (s)	7
Dose per frame (e-/ $Å^2$)	1.14
Accumulated dose (e-/$Å^2$)	45.7
Defocus range (- µm)	1.5-3.2
**Frames**	
Alignment software	MotionCor2
Frames used in final reconstruction	2-40
Dose weighting	Yes
**CTF**	
Fitting software	CryoSPARC Patch
Correction	full
**Particles**	
Picking software	CryoSPARC
Picked	1,617,236
Used in final reconstruction	306,475
**Alignment**	
Alignment software	CryoSPARC
Initial reference map	Ab initio
low pass filter limit (Å)	40
number of iterations	9
local frame drift correction	no
**Reconstruction**	
Reconstruction software	CryoSPARC
Box Size	316
Voxel size (Å)	0.829
Symmetry	C1
Resolution limit (Å)	1.658
Resolution estimate (Å)	3.28
Masking	Yes
Sharpening ($Å^2$)	-168
EMDB ID	EMD-16777
**Model Building**	
Number of protein residues	708
Number of DNA residues	254
Bond length outliers	0 out of 5593
Bond angle outliers	1 out of 7513
Bonds (R.M.S.D)	0.004
Angles (R.M.S.D)	0.571
CaBLAM outliers (%)	0.44
Ramachandaran favoured/allowed/outlier	98.27/1.73/0.00
Rotamer outliers (%)	0.34%
Clash score	8.77
Model vs Data CC (mask)	0.82
EMringer	1.34
FSC model vs map 0.5	0.261 (unmasked)
Molprobity score	1.29
PDB ID	8COM

Supplementary Table S2: DNA sequences used to wrap NCPs and the primers used to generate them.

**145 bp Widom-601 DNA**<br>5’-ATCAGAATCCCGGTGCCGAGGCCGCTCAATTGGTCGTAGACAGCTCTAGC<br>ACCGCTTAAACGCACGTACGCGCTGTCCCCCGCGTTTTAACCGCCAAGGGGATTACTCCCT<br>AGTCTCCAGGCACgTGTCAGATATATACATCGAT-3’<br><br>(PCR not used, see ‘Materials and Methods’)
**175 bp Widom-601 DNA (fluorescently-labelled)**<br>5’-ATGGAACACATTGCACAGGATGTATATATCTGACACGTGCCTGGAGACTA<br>GGGAGTAATCCCCTTGGCGGTTAAAACGCGGGGGACAGCGCGTACGTGCGTTTAAGCGGT<br>GCTAGAGCTGTCTACGACCAATTGAGCGGCCTCGGCACCGGGATTCTCCAGGGCGGCCGC<br>GTATT-3’<br><br>Forward primer: 5'-TAMRA-ATGGAACACATTGCACAGGATGTAT-3’<br>Reverse primer: 5’-6-FAM AATACGCGGCCGCCCTGGAG-3’
**147 bp *T. brucei* Centromere-Associated Repeat DNA**<br>5’-ATGCAATATGTAAGGTGTTTTGGTGTAAAACACGCATTCTTGCATAACAT<br>GCACAATGTGGCATGTTTGTGTGCAAATTGTGCACTATTGCGTATTTTCACGTCAAATACGCG<br>TTCATGCGTATGATTGCGCAAAAACAGTGTTGCA-3’<br><br>Forward primer: 5’-ATGCAATATGTAAGGTGTTTTGGTGT-3’<br>Reverse primer: 5’-TGCAACACTGTTTTTGCGCA-3’
**177 bp *T. brucei* Minichromosome Repeat DNA**<br>5’-CTAATAAATGGTTCTTATACGAATGAATATTAAACAATGCGCAGTTAACG<br>CTATTATACACAATAACTTTTAATGTGTGCAATATTAATTACAAGTGTGCAACATTAAATACAA<br>GTGTGTAACATTAATTTGCAAGTTTGCAACGCTGTTCTTTAGTGTTTAATGTGTGCAACAAAG-<br>3’<br><br>Forward primer: 5’- CTAATAAATGGTTCTTATACGAATGAATATTAAACAATGC-3’<br>Reverse primer: 5’- CTTTGTTGCACACATTAAACACTAAAGAAC-3’

References

1. Tan, Y.Z., Baldwin, P.R., Davis, J.H., Williamson, J.R., Potter, C.S., Carragher, B. and Lyumkis, D. (2017) Addressing preferred specimen orientation in single-particle cryo-EM through tilting. *Nat Methods*, **14**, 793- 796.
2. Ai, H., Sun, M., Liu, A., Sun, Z., Liu, T., Cao, L., Liang, L., Qu, Q., Li, Z., Deng, Z. et al. (2022) H2B Lys34 Ubiquitination Induces Nucleosome Distortion to Stimulate Dot1L Activity. Nat Chem Biol, 18, 972-980.
3. Vasudevan, D., Chua, E.Y.D. and Davey, C.A. (2010) Crystal Structures of Nucleosome Core Particles Containing the '601' Strong Positioning Sequence. Journal of Molecular Biology, 403, 1-10.
4. Hirai, S., Tomimatsu, K., Miyawaki-Kuwakado, A., Takizawa, Y., Komatsu, T., Tachibana, T., Fukushima, Y., Takeda, Y., Negishi, L., Kujirai, T. et al. (2022) Unusual nucleosome formation and transcriptome influence by the histone H3mm18 variant. Nucleic Acids Research, 50, 72-91.
5. Kono, H., Shirayama, K., Arimura, Y., Tachiwana, H. and Kurumizaka, H. (2015) Two Arginine Residues Suppress the Flexibility of Nucleosomal DNA in the Canonical Nucleosome Core. PLoS ONE, 10, e0120635.
6. Jumper, J., Evans, R., Pritzel, A., Green, T., Figurnov, M., Ronneberger, O., Tunyasuvunakool, K., Bates, R., Žídek, A., Potapenko, A. et al. (2021) Highly accurate protein structure prediction with AlphaFold. Nature, 596, 583-589.
7. White, C.L., Suto, R.K. and Luger, K. (2001) Structure of the yeast nucleosome core particle reveals fundamental changes in internucleosome interactions. The EMBO Journal, 20, 5207-5218.
8. Sato, S., Takizawa, Y., Hoshikawa, F., Dacher, M., Tanaka, H., Tachiwana, H., Kujirai, T., Iikura, Y., Ho, C.-H., Adachi, N. et al. (2021) Cryo-EM structure of the nucleosome core particle containing *Giardia lamblia* histones. Nucleic Acids Research, 49, 8934-8946.
9. Barbera, A.J., Chodaparambil, J.V., Kelley-Clarke, B., Joukov, V., Walter, J.C., Luger, K. and Kaye, K.M. (2006) The Nucleosomal Surface as a Docking Station for Kaposi's Sarcoma Herpesvirus LANA. Science, 311, 856-861.
10. Lesbats, P., Serrao, E., Maskell, D.P., Pye, V.E., O'Reilly, N., Lindemann, D., Engelman, A.N. and Cherepanov, P. (2017) Structural basis for spumavirus GAG tethering to chromatin. Proc. Natl. Acad. Sci. U.S.A., 114, 5509-5514.
11. Kraus, A.J., Vanselow, J.T., Lamer, S., Brink, B.G., Schlosser, A. and Siegel, T.N. (2020) Distinct roles for H4 and H2A.Z acetylation in RNA transcription in African trypanosomes. Nat Commun, 11, 1498.
12. Maree, J.P., Tvardovskiy, A., Ravnsborg, T., Jensen, O.N., Rudenko, G. and Patterton, H.-G. (2022) *Trypanosoma brucei* histones are heavily modified with combinatorial post-translational modifications and mark Pol II transcription start regions with hyperacetylated H2A. Nucleic Acids Research, 50, 9705-9723.
13. Min, J., Feng, Q., Li, Z., Zhang, Y. and Xu, R.-M. (2003) Structure of the Catalytic Domain of Human DOT1L, a Non-SET Domain Nucleosomal Histone Methyltransferase. Cell, 112, 711-723.
14. Worden, E.J., Hoffmann, N.A., Hicks, C.W. and Wolberger, C. (2019) Mechanism of Cross-talk between H2B Ubiquitination and H3 Methylation by Dot1L. Cell, 176, 1490-1501.e1412.
15. Anderson, C.J., Baird, M.R., Hsu, A., Barbour, E.H., Koyama, Y., Borgnia, M.J. and McGinty, R.K. (2019) Structural Basis for Recognition of Ubiquitylated Nucleosome by Dot1L Methyltransferase. Cell Reports, 26, 1681-1690.e1685.
16. Pettersen, E.F., Goddard, T.D., Huang, C.C., Meng, E.C., Couch, G.S., Croll, T.I., Morris, J.H. and Ferrin, T.E. (2021) UCSF ChimeraX: Structure visualization for researchers, educators, and developers. Protein Science, 30, 70-82.
